# Navigating T-Cell-Directed Therapies in Transformed Follicular Lymphoma

**DOI:** 10.3390/cancers18203391

**Published:** 2026-10-20

**Authors:** Viktoriya Zelikson, Reem Karmali

**Affiliations:** 1 Feinberg School of Medicine, Northwestern University, Chicago, IL 60611, USA; viktoriya.zelikson@nm.org; 2 Robert H. Lurie Comprehensive Cancer Center, Chicago, IL 60611, USA

**Keywords:** transformed follicular lymphoma, CAR-T, bispecific antibody

## Abstract

T-cell-engaging therapies have revolutionized care for relapsed or refractory large B-cell lymphoma. Transformed follicular lymphoma (tFL) has had limited representation in CAR-T or bispecific antibody trials. Thus, outcomes in this subgroup are not well-described. Furthermore, trials that include aggressive disease transformed from indolent lymphomas often report collective outcomes rather than results for individual histologic subsets despite biologic heterogeneity; this limits interpretability of results. Despite these limitations, there are consistent signals that tFL responds well to T-cell-engaging therapies. tFL has a unique tumor microenvironment, but it is not clear if or how this contributes to the favorable outcomes. More robust data are available for CAR-T compared to bispecifics in this population and differences in responsiveness between the two therapies are difficult to ascertain. More research is needed to better understand biologic interaction of tFL with T-cell-engaging therapies and response to therapy to inform future clinical trials.

## Simple Summary

Transformed follicular lymphoma (tFL) is a subtype of diffuse large B-cell lymphoma (DLBCL) that arises from follicular lymphoma. tFL displays aggressive disease biology and is managed with intensive chemotherapy. Relapses are common, and CAR-T and bispecifics, therapies which reprogram the immune system to recognize and target malignant B-cells, are often used. While much is known about outcomes of these therapies in DLBCL, less is known about their activity in the tFL subtype specifically. While there is no overwhelming evidence of tFL being an advantaged group among DLBCL subtypes when treated with T-cell-engaging therapies, some signals are encouraging. More data are needed to characterize CAR-T and bispecific efficacy in tFL.

Transformation of follicular lymphoma (FL) to a large B-cell lymphoma refers to the development of a large-cell lymphoma arising from FL and may be identified either concurrently or after a prior diagnosis of FL. The diagnosis is usually determined by pathology review, but clinically aggressive features, tempo of progression, and B symptoms suggest the diagnosis if biopsy is not feasible or indeterminate. The leading theory of transformation stems from the presence of common progenitor cells (CPC) that serve as a reservoir from which subclones may emerge [1]. The t(14;18) BCL-2 translocation is a key driver of cell survival, as it is in its indolent counterpart, but transformation lacks a single genetic event. Rather, it stems from a diverse spectrum of changes involving cell cycle, DNA damage repair, and immune evasion, among others [2].

Previously untreated transformed follicular lymphoma (tFL) has similar outcomes to de novo (dnDLBCL) with first-line chemoimmunotherapy. Those treated with chemotherapy for FL and those with early relapse have among the worst outcomes [3]. It is likely that aggressive disease biology and selection of chemotherapy-resistant clones contribute to this. These challenges are precisely why signals toward improved outcomes of tFL with T-cell-engaging therapy are welcomed.

In reviewing prospective and retrospective studies of T-cell-redirected therapies in tFL, interpretation of results is made challenging by several factors unique to the indolent transformed subset. These include results derived from exploratory sub-analyses of small populations which seldom reach statistical significance, lack of separation of tFL from other indolent transformed subsets in data reporting, and the potential for misclassification of histology without central review. Despite these limitations, it is valuable to look at recurring trends while being cautious not to overinterpret.

Representation of transformed indolent lymphoma in prospective CAR-T trials ranges from 11% to 22%; some trials report on tFL specifically, and others have grouped transformed indolent lymphoma subsets together. Table 1 summarizes trial results for CAR-T in transformed indolent lymphoma with details provided for tFL, where data are available. For CD19 CAR-T axicabtagene ciloleucel (axi-cel) with CD28 co-stimulatory domain, 5-year follow-up of ZUMA-1 in (third line) 3L+ showed a numerical difference in median event-free survival (EFS) in tFL compared to dnDLBCL (29.7 months (95% CI [2.3 months-not reached (NR)]) in tFL vs. 4.8 months (95% CI [3.0–7.3 months]) in dnDLBCL), though not statistically significant. In this study, tFL represented 16% of the cohort [4]. ZUMA-7 in second line (2L) included 11% of patients with tFL and showed similar outcomes for tFL vs. dnDLBCL, as reported in an early analysis [5]. The TRANSCEND study evaluated 4-1BB CD19 CAR-T lisocabtagene maraleucel (liso-cel) in 3L+ with 22% of its cohort consisting of tFL. Complete response (CR) rates in tFL vs. dnDLBCL were (63% (95% CI [49.3–75.6%]) vs. 49% (95% CI [40.0–57.7%]). However, median progression-free survival (mPFS) was improved for tFL vs. DLBCL (NR (95% CI [11.8 months-NR]) vs. 3 mo (95% CI [2.8–6.3 months)]) and there was a trend toward improved median overall survival (mOS) (NR (95% CI [21.1 months-NR]) vs. 12.7 months (95% CI [9–21.1 months])) [6].

Retrospective data mirror trial data and provide a more compelling argument for improved outcomes to CAR-T in tFL vs. dnDLBCL [10,11,12,13]. Table 2 summarizes relevant retrospective data. Cortese et al. noted superior 3 yr PFS (54% vs. 39%, *p* < 0.01) and OS (59% vs. 39%, *p* < 0.01) when comparing tFL to dnDLBCL. Patients with tFL and dnDLBCL possessed similar high-risk features such as prior lines of therapy, bulky disease, stage, etc. Some of these high-risk subgroups (DHL and relapsed/refractory (R/R) disease) fared better when originating from FL versus de novo disease [10]. In another study, Fabbri et al. grouped tFL and transformed marginal zone lymphoma (tMZL) when compared to dnDLBCL; tFL accounted for the majority of transformed diseases (90%). In this study, an improved 2 yr PFS (44% vs. 31%, *p* < 0.01) and OS (61% vs. 48%, *p* < 0.01) were reported for indolent transformed NHL (itNHL) vs. dnDLBCL. itNHL patients were more heavily pretreated and had higher incidence of exposure to prior autologous stem cell transplants (ASCTs) compared to dnDLBCL. When compared to tMZL directly, tFL had similar PFS and OS [11]. No comparative studies reported significant differences in cytokine release syndrome (CRS), immune effector-associated neurotoxicity syndrome (ICANS), or infections.

It is unclear why tFL may potentially respond better to CAR-T compared to dnDLBCL. On comparing the tumor microenvironment (TME) in FL pre- and post-transformation, T follicular helper cells are decreased, CD8+ cells are increased, and there is a higher expression of immunosuppressive markers such as PD-1 and LAG-3, coupled with impairment in effective crosstalk between the malignant B-cells and CD4/CD8 T-cells [14,15,16]. However, tFL tissue exhibits tumor infiltrating T, NK, macrophage, and dendritic cells that suggest there is at least active cell trafficking of these immune subsets into the tissue. Exploratory analyses in JULIET for 3L+ (tisa-cel) showed that while high CD3+ tumor infiltrate had better outcomes to CAR-T, high LAG3+CD3+ presence was detrimental [17]. How this relates to tFL histology is not clear. It is possible that CAR-T may be more effectively trafficked directly into the tumor tissue with the ability to overcome the local suppressive cytokine milieu; this hypothesis has yet to be confirmed but warrants evaluation. An exploratory analysis of TRANSCEND NHL 001 identified an “FL-like” gene-expression signature derived from genes differentially expressed between FL and DLBCL. A higher FL-like expression score was associated with longer PFS after liso-cel. The signature was not specific for histologically transformed FL and was elevated in some dnDLBCL samples but does underscore the importance of ongoing efforts to identify biologic predictors of response and survival with CAR-T in patients including those with tFL [18].

Although CAR-T has curative potential in both tFL and dnDLBCL, more is needed to improve outcomes. Augmenting CAR-T responses is of interest in tFL. Our group reported encouraging results with CAR-T and adjunctive Bruton tyrosine kinase inhibitor (BTKi) zanubrutinib in DLBCL [19]. Other trials have reported success, and a recent review summarizes the available evidence for combining BTK inhibitors with CAR T-cell therapy [20]. Another approach is to consider CAR-T in frontline to exploit healthier T-cells without exposure to prior chemotherapy. The ZUMA-23 trial is underway to evaluate CAR-T in high-risk 1L LBCL including tFL but limited to those without prior anthracycline therapy. Bi-and tri-specific CAR-T trials are also ongoing and include tFL with the expectation of yielding promising results in aggressive B-cell lymphoma collectively.

By contrast to CAR-T trials, trials evaluating bispecific antibodies (BsAbs) in aggressive B-cell lymphoma typically included fewer numbers of patients with tFL, as noted in Table 1. In the frontline setting, most BsAb trials underway have excluded this subset. In the R/R setting, granular data detailing outcomes in tFL subsets are more limited. Thus far, only two CD3/CD20 BsAbs, namely epcoritamab and glofitamab, have been approved in the United States for the treatment of DLBCL including transformed indolent lymphoma. The epcoritamab monotherapy trial (≥3L) sub-analysis showed that similar CR rates for all grouped transformed indolent lymphomas vs. dnDLBCL were 48% (95% CI [32–64%]) vs. 38% (95% CI [28–49%]) [7]. Glofitamab monotherapy (≥3L) results were reported for tFL specifically and included a significant number of patients treated with prior CAR-T. In the glofitamab study, CR rates for tFL vs. dnDLBCL were 41% (95% CI [18–67%]) vs. 36% (95% CI [26% vs. 48%]) [8].

In ≥2L, both these BsAb have been evaluated in combination with gemcitabine-oxaliplatin (GemOx) for patients who are ineligible for autologous stem cell transplantation. However, only the epcoritamab-GemOx trial included patients with transformed indolent lymphoma and reported a CR rate of 75% (95% CI [53–90%]) vs. 56% (95% CI [44–67%]) in transformed lymphoma vs. dnDLBCL [9]. Thus, it is uncertain whether the addition of chemotherapy, which is commonly used outside of the limitation of frailty, is beneficial in the tFL population.

When selecting between BsAb and CAR-T for tFL, relevant considerations include eligibility for CAR-T, time toxicity in cases of planned indefinite therapy, access to specialized cellular-therapy centers, and the maturity of available follow-up data. As the follow-up for CAR-T is longer and shows a steady plateau for relapse, it is the authors’ view that until more definitive and long-term data are presented for bispecifics, patients equally eligible should be preferentially offered CAR-T. As BsAb and CAR-T move into the frontline for DLBCL, data will invariably be extrapolated to tFL, which will impact sequencing. Retrospective data published by our group for DLBCL do not show a significant difference in outcomes based on sequencing of CAR-T and BsAbs but more work is needed to explore patient frailty influencing choice, T-cell exhaustion, and CAR-T-cell fitness post-BsAb [21].

An underexplored question of relevance to tFL in these reviewed studies is how bendamustine exposure impacts CAR-T and BsAb outcomes. As prior bendamustine use is a surrogate marker for prior lines of therapy and/or more aggressive disease in need of treatment, this topic is difficult to study retrospectively. DeCicco et al. reported inferior outcomes in FL treated with bendamustine within 1 year of CAR-T (2 yr OS 40% vs. 68%, *p* = 0.02) [22]. Other studies have noted a similar trend toward inferior outcomes to CAR-T or BsAb with recent bendamustine exposure in DLBCL [23]. Iacoboni et al. reported on bendamustine vs. non-bendamustine-exposed R/R DLBCL patients, noting significantly lower ALC at apheresis and significantly reduced PFS and OS that maintained the trend but lost significance in a matched analysis [24]. The impact of bendamustine surrounding T-cell-directed therapy continues to be debated and future studies assessing tFL outcomes should highlight this variable.

Transformed indolent lymphomas are heterogeneous in biology yet typically grouped together as a single entity or often excluded in clinical trials evaluating T-cell-redirecting therapy. Though repeated trends of improved outcomes are noted with CAR-T in trials and real-world data for tFL, robust numbers of these patients in prospective studies are lacking. While it does not eliminate unfavorable outcomes, CAR-T remains active in high-risk subgroups in tFL. Data are even more limited for BsAb in this subset. As ongoing trials evolve and new trials are designed, it will be important to enrich this group to generate more relevant data.

## Figures and Tables

**Table 1:** Prospective CAR-T and BsAb studies reporting outcomes in transformed lymphoma.

Trial	Product	Line of Therapy	Number of Patients in CART/BsAb Arm	Median Follow Up	% Transformed Patients	Comparator	Outcomes (Transformed vs. de Novo DLBCL)
CAR-T							
ZUMA-1 [4]	Axi-cel	≥3L	101	63 mo	16% tFL	tFL vs. DLBCL	mEFS 29.7 mo (95% CI [2.3 mo-NE]) vs. 4.8 mo (95% CI [3–7.3 mo])
ZUMA-7 [5]	Axi-cel	2L early R/R LBCL	180	25 mo	11% tFL	tFL vs. DLBCL	OR of overall response: 6.29 (0.98–62.11) vs. 5.58 (2.75–10.91) HR for event/death: 0.35 (0.16–0.77) vs. 0.37 (0.27–0.52) (OR and HR refer to axi-cel vs. SOC arm)
TRANSCEND NHL 001 [6]	Liso-cel	≥3L	269	18 mo	22% tFL	tFL vs. DLBCL	CR 63% (95% CI [49.3–75.6%]) vs. 49% (95% CI [40–57.7%]) mOS NR (95% CI [21.1 mo-NR]) vs. 12.7 mo (95% CI [9–21.1 mo]) mPFS NR (95% CI [11.8 mo-NR]) vs. 3 mo (95% CI [2.8–6.3 mo])
Bispecifics							
EPCORE NHL-1 [7]	Epcoritamab monotherapy	≥3L	157	11 mo	25% transformed DLBCL	Transformed DLBCL vs. DLBCL	CR 48% (95% CI [32–64%]) vs. 38% (95% CI [28–49%])
NP30179 [8]	Glofitamab monotherapy	≥3L	155	13 mo	18% tFL	tFL vs. DLBCL	CR 41% (95% CI [18–67%]) vs. 36% (95% CI [26–48%])
EPCORE NHL-2 [9]	Epcoritamab + GemOx	≥2L	103	13 mo	23% transformed DLBCL	Transformed DLBCL vs. DLBCL	CR 75% (95% CI [53–90%]) vs. 56% (95% CI [44–67%])

CR: complete response, HR: hazard ratio, tFL: transformed follicular lymphoma, DLBCL: diffuse large B-cell lymphoma, GemOx: gemcitabine/oxaliplatin, R/R: relapsed/refractory, mEFS: median event-free survival, mPFS: median progression-free survival, mOS: median overall survival, axi-cel: axicabtagene ciloleucel, liso-cel: lisocabtagene maraleucel, mo: months.

**Table 2:** Retrospective CAR-T studies reporting outcomes in transformed lymphoma.

Study	Number of Patients	Median Follow-Up	Demographic Differences (tFL vs. dnDLBCL *)	CR (tFL vs. dnDLBCL *)	PFS (tFL vs. dnDLBCL *)	OS (tFL vs. dnDLBCL *)	Adverse Events
Cortese et al. [10] ^+^ 14 US centers	691 (139 tFL)	29 mo	Prior bendamustine: 24% vs. 6% No difference in IPI, primary refractory disease, early relapse, need for bridging therapy, DH status, CAR-T product	45% vs. 31% (*p* < 0.01)	3 yr PFS 54% vs. 39% (*p* < 0.01) Primary refractory disease/early relapse of tFL vs. dnDLBCL: 3 yr PFS 46% vs. 35% (*p* = 0.04) DH arising from FL vs. de novo DH: 3 yr PFS 50% vs. 39% (*p* = 0.06)	3 yr OS 59% vs. 39% (*p* < 0.01) Primary refractory disease/early relapse of tFL vs. dnDLBCL: 3 yr OS 51% vs. 35% (*p* = 0.02) DH arising from FL vs. de novo DH: 3 yr OS 57% vs. 39% (*p* = 0.03)	“No difference in CRS or ICANS”
Fabbri et al. [11] Italian CART-SIE database	438 (107 with itNHL: 96 tFL, 11 tMZL)	22 mo	itNHL vs. dnDLBCL * Refractory to last line of therapy: 85% (86% in tFL, 55% in tMZL) vs. 73%, (*p* < 0.01) Prior ASCT: 46% vs. 24%, (*p* < 0.01) No difference in stage, IPI, bulky disease, median prior lines of therapy, response to bridging therapy, CAR-T product	itNHL vs. dnDLBCL * 62% vs. 53%	itNHL vs. dnDLBCL * 2 yr PFS 44% vs. 31% (*p* < 0.01) transformed FL vs. MZL mPFS: 17.2 mo (95% CI [6.5 mo–NR]) vs. 3.4 mo (95% CI [2.4 mo–NR])	itNHL vs. dnDLBCL * 2 yr OS 61% vs. 48% (*p* < 0.01) transformed FL vs. MZL mOS: 47 mo (95% CI [23.8 mo–NR]) vs. 16.6 mo (95% CI [3.2 mo–NR])	CRS any grade: 79% vs. 85% (*p* = 0.11) ≥Grade 3 CRS: 5% vs. 8% (*p* = 0.38) ICANS any grade: 19% vs. 20% (*p* = 0.78) ≥Grade 3 ICANS: 7% vs. 6% (*p* = 0.35) Infection: 10% vs. 17% (*p* = 0.09) ≥Grade 3 Infection: 6% vs. 5% (*p* = 0.13) ICAHT: 50% vs. 28%, *p* < 0.01 2 yr NRM: 11% vs. 4.1%, NS
Van der Galien et al. [12] ^+^ 7 CART centers in the Netherlands	272 (90 tFL)	29 mo	≥3 prior lines of tx: 47% vs. 12%, (*p* < 0.001) >6 mo since last therapy prior to CART: 24% vs. 14%, (*p* = 0.04) extranodal disease: 27% vs. 43%, (*p* = 0.002) Similar disease burden by LDH, bulky disease, and stage	82% vs. 66%	2 yr PFS 53% vs. 37% (*p* = 0.015)	2 yr OS 69% vs. 46% (*p* = 0.001))	“No significant differences in CRS or ICANS” CRS: 92% vs. 95% ≥Grade 3 CRS: 2% vs. 8% ICANS: 66% vs. 61% ≥Grade 3 ICANS: 26% vs. 23%
Thiruvengadam et al. [13] CIBMTR (2017–2023)	923	25 mo		63% (no comparator)	2 yr PFS 43% (no comparator)	2 yr OS 57% (no comparator)	CRS/ ≥ Grade 3 CRS: 85.3%/7.1% ICANS/ ≥ Grade 3 ICANS: 50.7%/21.6% 2 yr NRM: 9.1%

^+^ Abstract publication, * grouped transformed indolent lymphoma, CR: complete response, tFL: transformed follicular lymphoma, DLBCL: diffuse large B-cell lymphoma, PFS: progression-free survival, OS: overall survival, CRS: cytokine release syndrome, ICANS: immune effector-associated neurotoxicity syndrome, ICAHT: immune effector cell-associated hematotoxicity, IPI: international prognostic score, DH: double-hit lymphoma, tMZL: transformed marginal zone lymphoma, tx: therapy.

## Data Availability

No new data were created or analyzed in this study. Data sharing is not applicable.
